# Preparation of Polyester-Based Metal-Cross Linked Polymeric Composites as Novel Materials Resistant to Bacterial Adhesion and Biofilm Formation

**DOI:** 10.3390/molecules16010933

**Published:** 2011-01-21

**Authors:** Suhair Saleh, Bassam Sweileh, Sami O. Taha, Ruhaifa Mahmoud, Mutasem O. Taha

**Affiliations:** 1 Department of Pharmaceutical Sciences and Pharmaceutics, Applied Science University, Amman, Jordan; 2 Department of Chemistry, Faculty of Sciences, University of Jordan, Amman, Jordan; 3 Department of Biology, Faculty of Sciences, University of Jordan, Amman, Jordan; 4 Drug Discovery Unit, Department of Pharmaceutical Sciences, Faculty of Pharmacy, University of Jordan, Amman, Jordan

**Keywords:** bacterial biofilms, 2,6-pyridinedicarboxylate, copper complexes, silver complexes, polyesters

## Abstract

Bacterial biofilms constitute an extremely resistant form of bacterial colonization with dire health and economical implications. Towards achieving polymeric composites capable of resisting bacterial adhesion and biofilm formation, we prepared five 2,6-pyridinedicarboxylate-based polyesters employing five different diol monomers. The resulting polyesters were complexed with copper (II) or silver (I). The new polymers were characterized by proton and carbon nuclear magnetic resonance spectroscopy, inherent viscosity, infrared spectroscopy, differential scanning calorimetry and thermogravimetric analysis. The corresponding metal complexes were characterized by differential scanning calorimery and infrared spectroscopy. The amounts of complexed copper and silver were determined by atomic absorption spectrophotometry. Finally, the resulting composites were tested for their antibacterial potential and were found to effectively resist bacterial attachment and growth.

## 1. Introduction

Microorganisms are commonly found in close association with surfaces [1,2]. In the presence of moisture and nutrients, bacteria attach to surfaces and grow to form extensive films interspersed within extracellular polymer matrices [1,3]. Therefore, biofilms can be defined as matrix-enclosed bacterial populations adherent to each other and/or to surfaces or interfaces [1,2,4,5,6,7,8]. Compared to their single cell forms one significant consequence of bacteria growing as biofilms is their resistance to medical and industrial control strategies [1,2,8,9,10,11,12,13,14,15], which render biofilms of enormous impact on medicine and economy.

Biofilms can exist on many medical implants such as catheters, artificial hips, contact lenses, *etc.* and due to their increased resistance to antimicrobial agents, they can often only be treated by removal of the implant, which increases the trauma to the patient and the cost of treatment [1,4,15,16,17]. Two of the most problematic biofilm forming bacterial species are *Staphylococcus aureus* [18,19] and *Pseudomonas aeruginosa* [20]. Both strains are known to cause severe health and industry-related problems due to their resistant biofilms [5,18,19,20].

Bacterial biofilms have prompted, and are still prompting continuous efforts towards designing new materials capable of resisting biofilm formation at their surfaces [1,17]. Approaches to biofilm-resistant materials can be confined to few basic methods, namely, surface modification to reduce bacterial attachment and subsequent biofilm development [21,22,23,24,25], and impregnation of polymers with antimicrobials to prevent bacterial colonization [23]. Several approaches were used to release antimicrobials (e.g., ciprofloxacin) from device surfaces or to drive antimicrobials through the biofilm over a sustained period of time [23]. Many carrier systems were proposed, including biodegradable polymers such as poly(lactide-co-glycolide) and thermoreversible hydrogels [23,24,26,27,28,29,30] or metallic surfaces (particularly copper and silver) that release antibacterial ions over prolonged periods oftime [31,32,33]. For example, an epoxy resin-based coating containing micro-fine copper flakes and cured to a hard finish has been shown to have effective biocidal action and therefore can be of possible use to prevent biofouling in open waters as well as in enclosed environments [34]. On the other hand, copper tubing were found to inhibit biofilms particularly if Cu^++^ ions are periodically released in the water distribution system [35]. Moreover, bioceramics (e.g., hydroxyapatite and tricalcium phosphate) received recent interest as potential bone replacement materials due to their ability to resist bacterial colonization [36,37]. The great recent interest in designing new biofilm-resistant materials combined with the general lack of literature examples on biofilm-resistant surfaces based on metal-polymer complexes prompted us to suggest pyridine-based polyesters complexed with Cu^++^ or Ag^+^ as possible biofilm-resistant materials.

## 2. Theory

**Figure 1:**
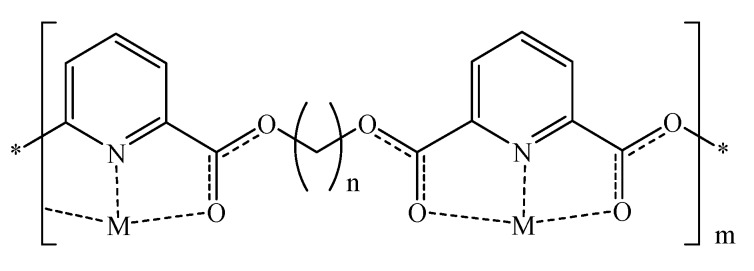
A schematic representation showing the basic idea of our proposed anti-biofilm polymeric-metal composites. n ranges from 2 to 6 atoms. M resembles metal cation: Cu^++^ or Ag^+^.

The potent antibacterial activities of silver and copper ions [38,39] led us to envisage anti-biofilm composite materials based on polymer/metal complexes capable of releasing trace amounts of silver or copper ions in a sustained manner. Such materials should maintain high surface metal concentrations capable of inhibiting bacterial biofilm formation. Copper- or silver-based composite materials should be superior to metal tubing as plastic materials are cheaper and easier to manipulate into different shapes and thicknesses. We were inclined to implement pyridine-based polyesters since 2,6-dicarbonylpyridine derivatives were reported to form stable metal complexes, e.g., with ruthenium, zinc or copper [40,41,42]. Furthermore, polyesters are known to possess enhanced thermal stabilities [43]. Figure 1 illustrates our proposed composite materials. According to our best knowledge, the use of metal complexes based on pyridine polyesters to generate surfaces resistant to bacterial biofilm formation is completely novel.

## 3. Results and Discussion

### 3.1. Preparation and Characterization of 2,6-Dicarboxypyridine Polymers

Five 2,6-dicarboxypyridine-based polymers were prepared by azeotropic condensation of five different diols with diethyl pyridine-2,6-dicarboxylate.

**Scheme 1:**
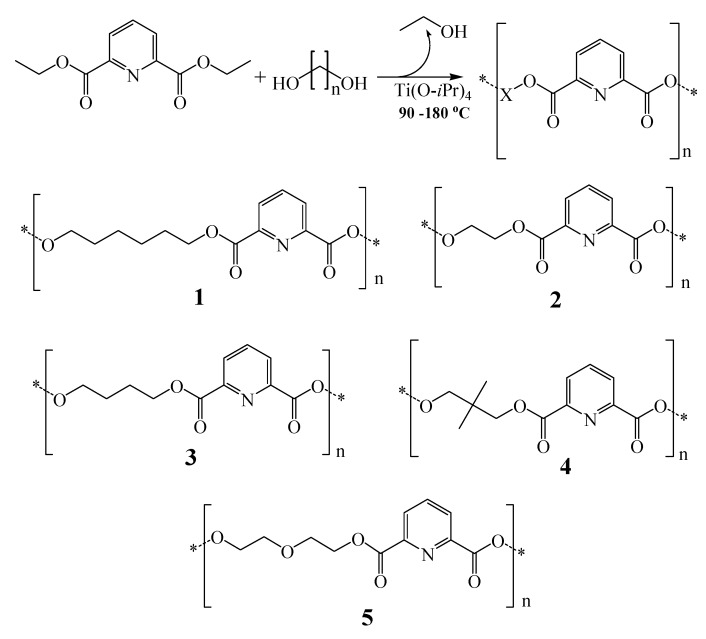
The condensation reaction and the prepared polymers: **1**: Poly(1,6-hexane-2,6-dicarboxylate pyridine); **2**: Poly(ethyleneglycol-2,6-dicarboxylate pyridine); **3**: Poly(1,4-butane-2,6-dicarboxylate pyridine); **4**: Poly(2,2-dimethyl-1,3-propane-2,6-dicarboxylate pyridine); **5**: Poly(diethyleneglycol-2,6-dicarboxylate pyridine); n ranges from 2 to 6 atoms.

To force the reactions to completion titanium tetraisopropoxide [Ti(O-*i*Pr)_4_] was added as Lewis acid catalyst, and reaction temperatures were raised to 180 °C to promote elimination of ethanol. Scheme 1 shows the condensation reactions and the prepared polymers. 

The polymers were characterized by inherent viscosity, differential scanning calorimetry (DSC), Fourier Transform Infra Red (FTIR) spectroscopy, proton and carbon nuclear magnetic resonance spectroscopy (^1^H-NMR and ^13^C-NMR), and thermogravimetric analysis (TGA). Table 1 shows the yields, major FTIR bands, inherent viscosities and glass transition points for the prepared polymers, while Table 2 shows their ^1^H-NMR and ^13^C-NMR signals.

**Table 1:** Prepared polymers, amounts of diols used in the preparation of each polymer, polymer yields, and select physicochemical properties.

Polymer ^a^		Diol		Yield	Ester C=O Stretching (cm^−1^)	Inherent Viscosity(g/dL)	Tg ^c^(°C)
No.	Name	Name	Amount (g) ^b^				
**1**	Poly(hexane-1,6-pyridine-2,6- dicarboxylate)	1,6-Hexanediol	1.18	60%	1741	0.303	8.3
**2**	Poly(ethyleneglycol pyridine 2,6-dicarboxylate)	Ethyleneglycol	0.62	73%	1729	0.301	74.7
**3**	Poly(butane-1,4-pyridine-2,6-dicarboxylate)	1,4-Butanediol	1.442	45%	1750	0.295	55.8
**4**	Poly(2,2-dimethyl-1,3-propane-	2,2-Dimethyl-1,3-propanediol	1.04	53%	1725	0.332	161.1
	pyridine-2,6-dicarboxylate)						
**5**	Poly(diethyleneglycol pyridine- 2,6-dicarboxylate)	Diethyleneglycol	1.06	32%	1728	0.288	33.1

^a^ See polymers in Scheme 1; ^b^ 10 mmol of each diol was condensed with 10 mmol diethyl pyridine-2,6-dicarboxylate. See Experimental section 4.3.2 Synthesis of polyesters. Each value represents diol weight in grams equivalent to 10 mmol; ^c^ Glass transition temperature.

**Table 2:** ^1^H- and ^13^C-NMR chemical shifts of the diethyl-2,6-pyridine dicarboxylate monomer and prepared polymers.

Polymer	^13^C-NMR shifts in CDCl_3_ (ppm)	^1^H-NMR shifts in CDCl_3_ (ppm)
**1**	25.6 (2 × CH_2_), 28.50 (2 × CH_2_), 66.10 (2 × CH_2_), 127.78 (2 × Aromatic CH), 138.25 (Aromatic CH), 148.30 (2 × Aromatic C), 164.48 (2 × C=O).	1.50 (m, 4H), 1.8 (m, 4H), 4.50 (m, 4H, CH_2_-O), 8.0 (m, 1H), 8.2 (m, 2H).
**2**	63.5 (4 × CH_2_), 128.36 (2 × Aromatic CH), 138.57 (Aromatic CH), 148.04 (2 × Aromatic C), 164.20 (2 x C=O).	4.50 (m, 4H, CH_2_-O), 8.0 (m, 1H), 8.2 (m, 2H).
**3**	25.31 (2 × CH_2_), 65.63 (2 × CH_2_), 127.90(2 × Aromatic CH), 138.30 (Aromatic CH), 148.49 (2 × Aromatic C), 164.51 (2 × C=O).	1.97 (m, 4H), 4.10 (m, 4H), 7.97 (m, 1H), 8.23 (m, 2H).
**4**	21.90 (2 × CH_3_), 35.49 (C), 70.41 (2 × CH_2_), 127.84 (2 × Aromatic CH), 138.24 (Aromatic CH), 148.32 (2 x Aromatic C), 164.28 (2 x C=O).	1.20 (s, 6H), 4.2 (s, 4H), 7.80 (m, 1H), 8.23 (m, 2H).
**5**	65.02 (2 × CH_2_), 68.91 (2 × CH_2_), 128.33(2 × Aromatic CH), 138.35 (Aromatic CH), 148.23 (2 × Aromatic C), 164.36 (2 × C=O).	3.9 (t, 4H), 4.54 (s, 4H), 7.920 (m, 1H), 8.23 (m, 2H).
Diethyl-2,6-dicarboxylate pyridine (monomer)	14.12 (2 x CH_3_), 62.16 (2 × CH_2_), 127.78 (2 × Aromatic CH), 138.25 (Aromatic CH), 148.49 (2 × Aromatic C), 164.48(2 × C=O).	1.20 (t, 2 × 3H), 4.4 (q, 2 × 2H), 7.9 (m, 1H), 8.2 (m, 2H).

Figure A in the Supporting Information shows the FTIR spectra of the five polymers aligned against each other. From the figure it is clear that the spectra of the polymers demonstrate intense characteristic bands ranging from 1,725–1,750 cm^−1^ corresponding to the stretching vibrations of the newly formed ester carbonyl functions. Table 2 lists the carbonyl stretching bands of the prepared polyesters.

Although the preparation conditions were identical for all prepared polymers the polymerization yields were rather variable and seemed to depend on the nature of the incorporated diol. Still, it is rather hard to simply correlate any diol property with the corresponding observed polymeric yield, which suggests that polymerization is influenced by several diol properties.

Unsurprisingly, polymers' ^ 13^C-NMR and ^1^H-NMR spectra (Table 2) exhibit aliphatic and aromatic resonance signals corresponding to the diol and pyridine-2,6-dicarboxylate fragments, respectively. Furthermore, emergence of ^13^C-NMR signal at 164 ppm further establishes ester formation across polymeric backbones, as shown in Scheme 1.

We determined the inherent viscosities of the polymers, as listed in Table 1, to assess their molecular size [44]. Clearly from the Table, all prepared polymers exhibited relatively high inherent viscosities, suggesting efficient polymerization reactions and significant molecular weights. In particular, polymer **4** [poly(2,2-dimethyl-1,3-propane-2,6-dicarboxylate pyridine] was characterized with the highest viscosity, while polymer **5** [poly(diethyleneglycol-2,6-dicarboxylate pyridine)] exhibited the least viscosity.

To further characterize the prepared polymers, we collected their DSC thermographs. DSC analyses reveal the response of any material to thermal challenge. Melting is normally expressed as downward endothermic broad band, while chemical degradation is usually expressed as an upward exothermic band. The transition from plastic to elastic states, *i.e.*, glass transition temperature or T_g_, is usually expressed as downward "step" on the DSC trait of a particular polymer [45]. Table 1 shows the T_g_ values of the prepared polymers, while Figure B in the Supporting Information shows their DSC traits.

Clearly from Table 1 and Figure B, polymer **4** has the highest T_g_ value of 161.1 °C while polymer **1** [poly(1,6-hexane-2,6-dicarboxylate pyridine)] exhibits the lowest value of 8.3 °C. The most reasonable explanation of this behavior is linked to the relative flexibilities of the polymeric scaffolds. The fact that polymer **1** includes 1,6-hexanediol units having seven rotatable bonds should promote polymeric flexibility, leading to significant reduction in T_g_ value, while the shorter 2,2-dimethyl-1,3-propanediol (four rotable bonds) is less flexible and therefore yields a rigid polymer of higher TG values. A similar trend exists among the other three polymers, *i.e.*, polymeric T_g_ is roughly inversely proportional to the number of rotatable bonds. 

Polymeric DSC traits unveil another interesting trend: Polymers **1**, **3** and **4** exhibit pronounced endothermic melting bands at 114, 180 and 278 °C, respectively. On the other hand, polymers **2** and **5** exhibit unusual thermal robustness, extending up to 300 °C. We believe this behavior is also related to the flexibility of the polymers. The increasing trend in melting temperatures of **1**, **3 **and **4** correlates well with the decreasing number of rotatable bonds in their respective diol monomeric fragments, *i.e.*, hexamethylene, tetramethylene and 2,2-dimethyl-1,3-propylene, respectively. Similarly, since polymer **2** possesses the shortest monomeric linker (ethylene) it is not unexpected for this polymer to exhibit robust thermal profile. However, we believe the impressive thermal stability of polymer **5** is related to the central ether oxygens of its diethyleneglycol monomeric linkers which seem to form dipole-dipole crosslinks within the polymeric matrix, thus elevating the melting point of the polymer.

To further assess polymeric chemical stability in response to thermal challenge we evaluated the thermogravimeric profiles (TGA) of the polymers. TGA assesses changes in mass upon exposure to heat. All prepared polymers illustrated good chemical stabilities up to 300 °C. Nevertheless, they degraded rather quickly upon exposure to higher temperatures. The TGA traits of the prepared polymers are shown in figure C in the Supporting Information.

### 3.2. Preparation and Characterization of Copper-Polymer and Silver-Polymer Composite films

To generate the corresponding silver and copper complexes, the prepared polymers were cast as thin films on glass cover slips. The resulting films were then softened at 200 °C and soaked in copper (II) sulfate (saturated) or silver nitrate (10% w/v) solutions. The solutions were maintained at 45 °C to enhance metal diffusibility through the polymeric films. Atomic absorption spectrophotometry was employed to determine the amounts of complexed metals in the resulting films. Table 3 shows the amounts of complexed copper or silver in different polymeric films. Experimental section 4.2.4 and Experimental section 4.3 provide complete details about the methodologies used.

**Table 3:** Metal content in different polymeric films as determined by atomic absorption spectrophotometry.

Polymer	Complexed Cu^+2^	Complexed Ag^+^
	(mg/g composite)*^a^*	(mg /g composite)*^ a^*
**1**	1.20 ± 0.07	2.9 ± 1.2
**2**	4.00 ± 0.10	13.7 ± 1.4
**3**	3.28 ± 0.13	2.3 ± 0.7
**4**	1.38 ± 0.10	1.2 ± 1.5
**5**	3.62 ± 0.13	3.0 ± 0.9

*^a^* Each value is the average of three measurements ± 1 standard deviation.

Clearly from Table 3, polymers **2 **and **5 **showed high capacities in capturing metal ions (*i.e.*, copper and silver). Polymer **2**, however, illustrated the highest affinity to both metal ions, particularly silver. Apparently, **2** possesses an optimal diol length to accommodate metal ions within the chelating pyridine dicarboxylate units. On the other hand, the relatively high chelating efficiency of polymer **5** can be attributed to extra metal chelation power due to the extra ether oxygen atoms in the diethyleneglycol monomeric units.

To further probe the complexation process, we decided to assess the DSC and FTIR profiles of the polymer-metal matrices. Copper- and silver-complexed polymeric matrices were prepared by vigorously mixing saturated methanolic metal solutions with saturated polymeric chloroform solutions. The resulting precipitates were assessed by FTIR and DSC.

#### 3.2.1. Spectroscopic and calorimetric characterization of copper-polymer complexes

The FTIR traits of the polymer-copper complexes revealed variable shifts in the carbonyl stretching vibrations upon complexaion. Table 4 illustrates the shifts in polymeric carbonyl stretching vibrations upon complexation to copper, while figure D in Supporting Information shows the FTIR spectrums of copper complexes of the five polymers.

**Table 4:** Characteristic carbonyl stretching vibrations of each polymer.

	Ester Carbonyl Stretching Band*^b^* (cm^−1^)		
Polymer	Before Complexaion	Cu^2+^ Complexes	Ag^2+^ Complexes
**1**	1741	1744	1722
**2**	1729	1711	1732
**3**	1750	1737	1723
**4**	1725	1737	-----
**5**	1728	1711	1734

*^a^* Polymers as in Scheme 1; *^b^* The corresponding infrared charts are shown figure D in the Supporting Information.

Clearly from Table 4, polymer **1** exhibited negligible shift in its carbonyl stretching band upon complexation to copper ions, suggesting a minimal degree of carbonyl-mediated copper complexation in this case, which agrees with the relatively low copper levels in this complex as measured by atomic absorption (Table 3). On the other hand, polymers **2**, **3** and **5** illustrated significant downward shifts in their carbonyl stretching bands, indicating the formation of significant levels of copper complexes, which also agrees with their higher copper contents in Table 3.

**Scheme 2:**
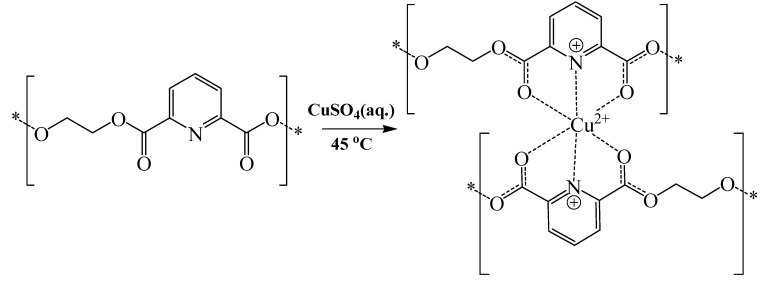
Schematic representation illustrating the proposed complexation of polymer **2** to copper.

**Scheme 3:**
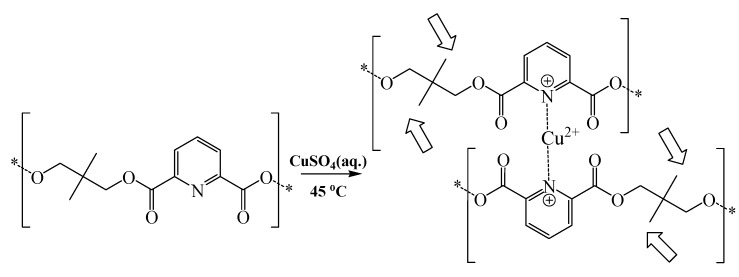
Schematic representation illustrating the proposed copper complex with polymer **4**. Broad arrows point to methyl groups believed to sterically hinder the complexation of copper ions to polymeric ester carbonyls.

Strangely, the carbonyl stretching band of polymer **4** illustrated a significant upward shift upon complexation to copper. The most probable explanation for this behavior is related to the steric constrains imposed by the 2,2-dimethyl groups, which seem to hinder carbonyl coordination to copper ions, and therefore leaves the pyridine nitrogen as sole electron donor in the coordination complex. This effect seems to enhance the double bond character of the carbonyl groups and therefore increases their stretching vibrations, as illustrated in Scheme 3.

Copper complexation caused significant alterations in the DSC traits of all five polymers. Table 5 lists major endothermic and exothermic bands observed for the polymers before and after complexation with copper, while figure E in Supporting Information shows the corresponding DSC charts. Apparently, copper complexation rendered endothermic bands of polymers **1**, **2**, **3** and **4** sharper, which is probably attributed to the enthalpic homogeneity of the newly formed metal-ligand coordinate bonds compared to the heterogeneity of attractive interactions existing within polymeric matrices prior to complexation (e.g., hydrogen-bonding, diapole-diapole interactions, *etc.*). Interestingly, the slight Tg step seen in the DSC trait of polymer **5** converted into deep endothermic band at 173 °C upon complexation to copper, which is indicative of extensive polymeric crosslinking due to metal complexation.

**Table 5:** Characteristic DSC bands of polymeric-copper composites.

	Endothermic bands (°C) *^b^*		Exothermic bands (°C) *^b^*	
Polymer*^a^*	Before Complexation	After Copper Complexation	Before Complexation	After Copper Complexation
**1**	114*^ f^*	242*^ f^*	73*^ f^*	270*^ f^*
**2**	77 *^c,d,e^*	260*^ f^*	---	---
**3**	180*^f^*	180*^ f^*	---	---
**4**	260	170*^ f^*	---	---
**5**	---	173	---	---

*^a^* Polymer numbers as in Scheme 1; *^b^* The corresponding DSC charts are shown in figure E in the Supporting Information;*^c^* Broad band; *^d ^*Related to transition glass; *^e ^*Shallow; *^f^* Intense and sharp.

Interestingly, the endothermic bands of polymers **1** and **2** shifted significantly upwards upon polymer-copper complexation, while polymer **3** maintained its endothermic band at 180 °C upon complexation. On the other hand, polymer **4** showed a significant downward shift in its endothermic band after complexation to copper. We believe these apparently contradictory behaviors can be attributed to the competing effects of polymeric flexibility and polymeric crosslinking by copper ions. Copper complexation, although minimal in polymer **1** (as deduced earlier from FTIR and copper loading levels), seems to significantly limit its polymeric flexibility causing the apparent upward shift in the endothermic band. A similar explanation is applicable in the case of polymer **2**, albeit more pronounced than polymer **1**. In case of polymer **3**, it seems that the uncomplexed polymer exhibits comparable polymeric flexibility to the crosslinked version, which explains the small change in the position of the endothermic band upon copper complexation. 

The thermal behavior of polymer **4** might be explained based on the limited flexibility of the uncomplexed polymeric backbone due to the 2,2-dimethyl substituents, which seems to cause high-temperature endothermic bands seen in the uncomplexed polymer case. On the other hand, copper-complexation seems to disturb existing attractive interactions within the polymeric matrix leading to downward shift in the corresponding endothermic band.

Regarding the exothermic bands seen in the DSC of polymer **1**, apparently, the relatively low temperature of the exothermic band seen before copper complexation (at 73 °C) suggests it is related to polymeric rearrangement rather than degradation. However, upon copper-crosslinking, this band was replaced by another exothermic degradation band at 270 °C.

#### 3.2.2. Spectroscopic and calorimetric characterization of silver-polymer complexes

The FTIR traits of the polymer-silver complexes revealed different carbonyl stretching shifts compared to copper complexation. Table 4 compares the shifts in polymeric carbonyl stretching vibrations upon complexation to copper and silver, while figures D and F in the Supporting Information show the FTIR spectrums of the copper and silver complexes of the five polymers. 

Clearly from Table 4, polymers **2** and **5** exhibited negligible shifts in carbonyl stretching bands upon complexation to silver ions suggesting a minimal degree of carbonyl-mediated coordination to silver, which contradicts the high silver contents of their complexes as measured by atomic absorption (particularly polymer **2**, Table 3). Apparently, polymers **2** and **5** bind to silver ions via their pyridine nitrogen atoms thus causing minimal shifts in the carbonyl stretching bands, reminiscent of copper complexation to polymer **4** (Scheme 3). This proposition is supported by the pronounced changes seen in the DSC profiles of polymers **2** and **5** upon complexation with silver (Table 6 and figure G in the Supporting Information). On the other hand, carbonyl stretching bands of polymers **1** and **3** showed a significant downward shifts upon complexation to silver. Apparently, silver ions coordinate to the carbonyl groups of these polymers causing significant reduction in their double bond character, a similar scenario to that shown in Scheme 2. The complexations are clearly evident from the significant alteration in the DSC profiles of polymers **1** and **3** upon complexation to silver (Figures B and G in the Supporting Information).

**Table 6:** Characteristic DSC bands of polymeric-silver composites.

	Endothermic bands (^o^C) *^b^*		Exothermic bands (^o^C) *^b^*	
Polymer *^a^*	Before Complexation	After silver Complextaion	Before Complexation	After silverComplexation
**1**	114*^ f^*	138 *^c,e^*	73*^ f^*	204 *^f^*
**2**	77 *^c,d,e^*	111 *^c,e^*	---	222 *^f^*, 274*^f^*
**3**	180*^f^*	---	---	267 *^f^*
**4**	260	---	---	180 *^c,e^*
**5**	---	163 *^c,e^*	---	210 *^f^*

*^a^* Polymer numbers as in Scheme 1; *^b^* The corresponding DSC charts are shown in figure G in the Supporting Information; *^c^* Broad band; *^d^* Related to transition glass; *^e ^*Shallow; *^f ^*Intense and sharp.

Strangely, the carbonyl stretching band of polymer **4** disappeared completely upon complexation to silver. We believe this behavior is caused silver-mediated degradation of this polymer through which the pyridine-dicarboxylate units loose their carboxylic acid groups. DSC trait of polymer **4** supports this conclusion: it lacks any significant endothermic or exothermic bands, in stark contrast to the DSC profiles of other polymers.

However, silver complexation caused significant alterations in the DSC traits of the remaining four polymers. Table 6 lists major endothermic and exothermic bands observed for the polymers before and after complexation with silver, while figure G in the Supporting Information shows the corresponding DSC charts. Silver complexation shortened the endothermic bands of polymers **1**, **2**, **3** and **5**, which is probably attributed to disruption of existing inter-polymer interactions (e.g., hydrogen-bonding, diapole-diapole interactions, *etc.*) upon complexation with silver ions. Most notably, the silver complexes of these polymers (*i.e.*, **1**, **2**, **3** and **5**) exhibit intense sharp exothermic bands above (or at) 200 °C. We believe this behavior is related to certain three-dimensional rearrangement of the polymer-silver composite matrix upon heating.

### 3.3. The Antibacterial Properties of Copper-Polymer and Silver-Polymer films

Thin composite films were prepared from copper- and silver-based polymeric complexes (see Experimental section 4.3.3. and Experimental section 4.3.4). Subsequently, they were challenged with biofilm-forming *Pseudomonas* bacteria (see Experimental section 4.4 for more details). Table 7 shows the number of bacterial colonies that were attached to different polymeric films after 2 h [49] of incubation in concentrated bacterial suspension (see Experimental section 4.4.3).

Clearly from Table 7, both silver- and copper-complexed polymers diplayed significant anti-biofilm properties compared to uncoated glass cover slips. Unsurprisingly, silver-based polymers seemed superior to copper analogues, which agrees with the findings of Harrison *et al.* [46], *i.e.*, silver is one of the most toxic metals on different microorganisms including the *Pseudomonas* strain used in the current study. However, this trend seems to be reversed in the case of silver complex of polymer **2**.

**Table 7:** Nnumber of attached bacteria to composite polymer-metal films after 2 h of incubation in 1 × 10^5^ - 1 × 10^6 ^CFU ^a^/mL.

	Average Bacterial Colonization Density	
	(CFU *^a^*/cm^2^) ± Standard Deviation	
Polymers	Ag-Complex	Cu-complex
1	0.7 ± 0.6	11 ± 5
2	40 ± 11.4	5 ± 5
3	0.8 ± 0.5 *^b^*	0.3 ± 0.4 *^b^*
4	0.4 ± 0.4 *^b^*	0.2 ± 0.4 *^b^*
5	1.6 ± 1.2	4 ± 6
**Glass cover slips **	2440.1 ± 279.9	

*^a^* Colony forming units, the values represent the average of three readings; *^b^* No significant growth.

In an attempt to explain the behavior of polymer **2**, we studied the amounts of released silver from each composite film after incubation in microbial media over 24 h. Table 8 summarizes the results. Clearly, polymer **2** showed the highest levels of released silver over this time interval, suggesting that high polymeric metal leaching rates coincided with inferior anti-biofilm polymeric properties. This behavior can be explained as follows: Leached metal ions tend to suppress planktonic bacteria prompting bacteria to form biofilms via triggering SOS response [47]. In fact, biofilms are known to resist intoxication by heavy metals in minimal media [17,48]. Furthermore, silver leaching reduces the amount of silver within the vicinity of polymeric surface, which should further encourage bacterial growth at polymeric surface.

**Table 8:** Released Ag^+^ in microbial testing media.

Polymer	Released Ag^+^(ppm) from 1 mg composite After 24 h *^a^*
**1**	1.3 ± 1.1
**2**	4.2 ± 1.7
**3**	1.8 ± 2.4
**4**	0.9 ± 2.0
**5**	2.6 ± 1.2

^a^ Each value represent the average of three measurements ± standard deviation.

These explanations are supported by the anti-biofilm behavior of the silver complex of polymer **4**, which combined the best anti-biofilm behavior and least silver release rates. Overall, it seems that effective polymeric resistance to biofilm formation requires slow-release of metal ions at the polymeric surface to guarantee high concentration of biocidal metal within the vicinity of the polymeric surface while minimizing the concentration of the metal within the bulk of the growth medium.

## 3. Conclusions

Towards achieving polymeric films of anti-biofilm properties, five pyridinedicarboxylate-based polyesters were prepared and complexed with copper (II) or silver ions. The polymers were characterized by proton and carbon nuclear magnetic resonance spectroscopy, inherent viscosity, infrared spectroscopy, differential scanning calorimery and thermogravimetric analysis. The metal complexes were characterized by differential scanning calorimery and infrared spectroscopy. The amounts of complexed metals were determined by atomic absorption. The resulting composites were tested for their antibiofilm potential and were found to effectively resist bacterial attachment and colonization. Poly(2,2-dimethyl-1,3-propylene-pyridine-2,6-dicarboxylate) (**4**) showed the best anti-biofilm results and least metal leaching rates. This project points to the potentialvutility of metal-complexed pyridine-based polyesters for the manufacture of potent anti-biofilm polymers.

## 4. Experimental

### 4.1. Materials

Commercially available analytical grade reagents were used without further purification. Chemicals were purchased from the appropriate commercial sources: 2,6-pyridinedicarboxylic acid, 2,2-dimethyl-1,3-propanediol, 1,4-butanediol, 1,6-hexanediol, ethyleneglycol, diethyleneglycol, anhydrous sodium sulfate, and sodium bicarbonate from Acros Organics (USA); titanum tetraisopropoxide Ti(O-*i*Pr)_4_, nitric acid and sulfuric acid from Scharlau (Spain), copper sulphate from Sigma (USA), silver nitrate from Sigma (USA), chloroform, absolute ethanol, methanol, diethyl ether from Merck (USA).

### 4.2. Measurements and Instrumentation

#### 4.2.1. FTIR spectroscopy

The FTIR spectra (from 500-4000 cm^−1^) of the monomers and polymers were recorded as neat films using a Thermo Nicolet Nexus 670 FT-IR spectrophotometer (Madison, WI, USA). The films were prepared by cast solution of purified monomer or polymer in chloroform over NaCl plates. The solvent was evaporated and further dried under vacuum. For preparing polymer-copper films for FTIR analysis, a chloroform solution (3 mL) of the particular polymer (50 mg) was added dropwise to saturated methanolic copper sulfate solution under vigorous stirring at ambient temperature. Subsequently, the resulting precipitate was filtered and dried under vacuum and ambient temperature to yield greenish white powder. The powdered complex was later used to prepare KBr discs for FTIR analysis.

#### 4.2.2. NMR spectroscopy

The ^1^H-NMR and ^13^C-NMR spectra of the monomers and polymers were recorded on a 300 MHz Bruker avance DPX 300 Spectrometer (Wissembourg Cedex, France) in deuterated chloroform. Chemical shifts (δ) are given in ppm with tetramethylsilane (TMS) as the internal standard. 

#### 4.2.3. Elemental analysis

The determination of the amounts of complexed alkylthiols in polymeric films was conducted using a Perkin-Elmer Euro-Vector 8910 Elemental Analyzer. 

#### 4.2.4. Atomic absorption

Concentrations of the metal ions were determined with a Varian-Spectra A 250 Plus (USA) atomic absorption spectrophotometer. Five standard copper (II) sulfate and silver nitrate analytical grade solutions were prepared in deionized water to construct appropriate calibration curve: 100, 150, 200, 250, and 300 ppm. 

*Quantification of Complexed Metals on Polymer Films.* The metal-containing films were pealed off from the casting plates. Thereafter, 20 mg of the polymeric-composites were collected and suspended in concentrated nitric acid (10 mL). The suspension was shaken for 20 min, and sonicated for additional 10 min, filtered and assayed. Filtered deionized water was employed as blank.

*Determination of released metals in the bacteria growth media.* The metal-complexed polymeric films were left in phosphate buffer solution (PBS) bacterial growth media (15 mL) over 24 h. Subsequently, the buffer solution was collected, concentrated to *ca.* 10 mL in the fume cupboard, transferred to a 10 mL-volumetric flask and completed to the mark using deionized distilled water. The solution was assayed employing a Varian-Spectra A250 Plus (USA) atomic absorption spectrophotometer. Deionized water was employed as blank.

#### 4.2.5. Polymer solution viscosity

The inherent viscosities were determined for the corresponding polymeric solutions in chloroform (0.5%w/v) employing Ubbelhode glass capillary viscometer (Rheotek, Poulten Selfe & Lee Ltd., Essex, England) in thermostated water bath temperature controlled at 25 ± 0.1 °C. The polymeric solutions were temperature equilibrated for approximately 15 min before measuring viscosity. Measurements were repeated several times until reproducible values were obtained. 

#### 4.2.6. Thermal analyses

*Glass transition temperature* (T_g_) of polymer samples were studied with a Netzsch DSC 204 F1 Differential Scanning Calorimeter (Selb Bavaria, Germany). T_g_ measurements, done by differential scanning calorimetry (DSC), were conducted on 10 ± 2 mg samples under a dry nitrogen atmosphere. The samples were first heated from ambient temperature to 180 °C and maintained for 2 min before rapid cooling with liquid nitrogen to the start temperature. The thermal behaviors of the samples were probed by heating the molten state at a heating rate of 10 °C/min. The T_g_ values were taken as the mid point of the step transition.

For preparing polymer-metal films for DSC analysis, a chloroform solution (3 mL) of the particular polymer (50 mg) was added drop wise to saturated copper sulfate or silver nitrate methanolic solution under vigorous stirring at ambient temperature. The resulting precipitate was filtered and dried under vacuum and ambient temperature to yield greenish white powder. This powdered complex was later used for DSC analysis. 

Thermal Stability: The thermal stabilities of the samples were studied by thermogravimetric analysis (TGA) with a Netzsch STA 409 PG/PC thermal analyzer (Selb Bavaria, Germany). Measurements were conducted at a heating rate of 20 °C/min under a dry nitrogen atmosphere purging at a flow rate of 50 mL/min.

### 4.3. Synthesis

#### 4.3.1. Synthesis of diethyl pyridine-2,6-dicarboxylate

A magnetically stirred solution of 2,6-pyridinedicarboxylic acid (3.0 g, 18 mmol) in dry ethanol(50 mL) was refluxed with concentrated H_2_SO_4_ (3 mL, 98%) for 24 h. The reaction was terminated by cooling to room temperature and quenching with saturated sodium bicarbonate aqueous solution(30 mL). The mixture was then extracted with chloroform (3 × 20 mL). The organic layers were combined and dried with anhydrous MgSO_4_. The organic solvent was removed *in vacuu* to leave diethyl 2,6-pyridinedicarboxylate as white crystalline solid, mp: 46 °C, Yield: 84%.

#### 4.3.2. Synthesis of polyesters

Diethyl pyridine-2,6-dicarboxylate (10 mmol), the corresponding diol (10 mmol, amounts as given in Table 1), and titanium tetraisopropoxide [Ti(O-*i*Pr)_4_] catalyst (5%w/w) were introduced into a glass reaction tube (150 × 30 mm i.d.) equipped with a gas inlet and outlet and stopcocks. Subsequently, the reaction mixture was quickly heated to 90 °C under a stream of nitrogen gas. Thereafter, the reaction temperature was gradually raised (15 °C every 30 min) until 180 °C. Subsequently, the nitrogen stream was replaced by vacuum pump under which the reaction mixture was maintained at 180 °C for 1 h. Finally, the reaction mixture was allowed to cool down to ambient temperature to yield solid polymeric materials that were dissolved in 40 mL of chloroform and filtered. The solution was then purified by repetitive dissolution in chloroform (40 mL), filtration and precipitation in methanol (150 mL). Solvent traces were removed by keeping the polymer under vacuum at 80 °C for 2 h. The yields of the prepared polymers were in the range 32–73%. 

#### 4.3.3. Preparation of polymer films

Polymeric films were prepared by dissolving the polymer (25 mg) in chloroform (15 mL) and casting on a stainless steel (or glass) plate (2.2 cm × 2.2 cm), such that the polymeric solution covered the whole plate. The solvent was allowed to evaporate over 1 h at ambient temperature. Subsequently, polymer-covered plates were heated to 180 °C under vacuum for 5 min.

#### 4.3.4 Preparation of polymer-metal composite films

The polymer films were soaked in aqueous copper sulfate (100 mL, saturated) or silver nitrate solution (100 mL, 10% w/v) at 45 °C over 24 h or 48 h, respectively. Subsequently, they were removed from the metal solutions and allowed to stand at ambient temperature over 1 h. Thereafter, the films were washed with distilled water (20 mL) and dried under ambient conditions.

### 4.4. Microbiological Evaluation

#### 4.4.1. Bacterial culture and media

*Pseudomonas aeruginosa ATCC 27853* was used in this study. The bacteria were stored at −20 °C in glycerol. Before testing, frozen bacteria was initially inoculated onto Tryptic Soy Agar (TSA) plates and incubated at 37 °C over 24 h. However, to prepare a working culture, Phosphate Buffer Saline (PBS, pH 7.4; NaCl: 8.0g/L; KCl: 0.2g/L; Na_2_HPO_4_: 1.15g/L; KH_2_PO_4_: 0.2g/L) was inoculated with a full loop of TSA-cultured bacteria and kept in a shaker-incubator for 16 h at 37 °C.

#### 4.4.2. Preparation of test surfaces

Glass cover slips (22 mm × 22 mm) were washed with detergent, rinsed with distilled water and immersed in concentrated nitric acid for 5 min. After five consecutive rinses in distilled water (50 mL), the glasses were dried and sterilized by autoclaving. The coated cover slips were immersed in 70% ethylalcohol for 1 min just before testing.

#### 4.4.3. Evaluation of polymeric resistance against bacterial biofilm formation

An overnight TSB-grown 10^8^ cfu/mL (colony forming unit/mL) culture of *Pseudomonas aeruginosa* was diluted by a factor of 1:100 with PBS to give a final concentration of 10^5^ to 10^6^ cfu/mL. Subsequently, 15 mL-aliquots of the resulting bacterial suspension were introduced into 50-mL tubes each containing a single, vertically positioned, coated or uncoated glass cover slip to test their tendency to support the formation of bacterial biofilms. All tubes were incubated at room temperature with shaking (100 rpm) for 2 h. Subsequently, the coated cover slips were removed and aseptically placed in tubes containing 10 mL sterile PBS and shaken vigorously for 10 min, then rinsed with three successive 10 mL-volumes of PBS to remove loosely attached bacteria. Shake and rinses of each polymer were pooled and 100 μL were spread on TSA and incubated for 16 h at 37 °C.

After rinsing, each tile was removed with forceps and gently placed flat on the surface of TSA plate. After 1 min, the cover slip was transferred to a second plate and the first plate spread with a glass spreader. The process was repeated through a succession of 15 TSA plates to assess tightly attached bacteria. Number of attached bacteria was determined by counting the colony forming units (CFU) after 16 h of incubation at 37 °C.

## Supplementary Materials

Supplementary materials’ can be accessed at http://www.mdpi.com/1420-3049/16/1/933/s1.
